# The green ash transcriptome and identification of genes responding to abiotic and biotic stresses

**DOI:** 10.1186/s12864-016-3052-0

**Published:** 2016-09-02

**Authors:** Thomas Lane, Teodora Best, Nicole Zembower, Jack Davitt, Nathan Henry, Yi Xu, Jennifer Koch, Haiying Liang, John McGraw, Stephan Schuster, Donghwan Shim, Mark V. Coggeshall, John E. Carlson, Margaret E. Staton

**Affiliations:** 1 Department of Entomology and Plant Pathology, University of Tennessee, Knoxville, TN 37966 USA; 2 Department of Ecosystem Science and Management, Pennsylvania State University, University Park, PA 16802 USA; 3 Department of Plant Biology and Pathology, Rutgers University, New Brunswick, NJ 08901 USA; 4 Department of Genetics and Biochemistry, Clemson University, Clemson, SC 29634 USA; 5 Northern Research Station, USDA Forest Service, Delaware, OH 43015 USA; 6 Center for Comparative Genomics and Bioinformatics, Pennsylvania State University, University Park, PA 16802 USA; 7 Department of Forestry, University of Missouri, Columbia, MO 65211 USA

**Keywords:** Transcriptome, RNASeq, Assembly, *Fraxinus*, Emerald ash borer, Heat, Drought, Cold, Ozone, Stress response

## Abstract

**Background:**

To develop a set of transcriptome sequences to support research on environmental stress responses in green ash (*Fraxinus pennsylvanica*), we undertook deep RNA sequencing of green ash tissues under various stress treatments. The treatments, including emerald ash borer (EAB) feeding, heat, drought, cold and ozone, were selected to mimic the increasing threats of climate change and invasive pests faced by green ash across its native habitat.

**Results:**

We report the generation and assembly of RNA sequences from 55 green ash samples into 107,611 putative unique transcripts (PUTs). 52,899 open reading frames were identified. Functional annotation of the PUTs by comparison to the Uniprot protein database identified matches for 63 % of transcripts and for 98 % of transcripts with ORFs. Further functional annotation identified conserved protein domains and assigned gene ontology terms to the PUTs. Examination of transcript expression across different RNA libraries revealed that expression patterns clustered based on tissues regardless of stress treatment. The transcripts from stress treatments were further examined to identify differential expression. Tens to hundreds of differentially expressed PUTs were identified for each stress treatment. A set of 109 PUTs were found to be consistently up or down regulated across three or more different stress treatments, representing basal stress response candidate genes in green ash. In addition, 1956 simple sequence repeats were identified in the PUTs, of which we identified 465 high quality DNA markers and designed flanking PCR primers.

**Conclusions:**

North American native ash trees have suffered extensive mortality due to EAB infestation, creating a need to breed or select for resistant green ash genotypes. Stress from climate change is an additional concern for longevity of native ash populations. The use of genomics could accelerate management efforts. The green ash transcriptome we have developed provides important sequence information, genetic markers and stress-response candidate genes.

**Electronic supplementary material:**

The online version of this article (doi:10.1186/s12864-016-3052-0) contains supplementary material, which is available to authorized users.

## Background

Green ash (*Fraxinus pennsylvanica* Marsh.) is the most widely distributed species in the *Fraxinus* genus in North America. Green ash is valuable both economically and ecologically. Green ash produces a large number of seeds, an important source of food for a diverse array of wildlife species [1]. It has been widely planted as a street tree, in parks, and in residential areas due to fast growth and adaptability to urban conditions. Both natural stands and urban plantings of green ash are now seriously threatened by the emerald ash borer (EAB, *Agrilus planipennis* Fairmaire), a pest of Asian ash species accidentally introduced into North America [2]. EAB was originally identified as the cause of widespread death and decline of native ash trees in Michigan and Ontario in 2002 [2]. EAB has since spread quickly and is currently found in 26 U.S. states and two Canadian provinces [3]. All native North American ash trees are considered susceptible to this pest, and mortality rates of up to 99 % have been observed in forest stands 6 years after infestation [4]. EAB has killed millions of ash trees in Michigan, Ohio and Indiana, and is spreading rapidly across North America [5]. Economic and ecological damage is expected to occur as the pest spreads [6–8] with estimates of costs due to lost tree value, removal and replacement ranging from $10.7 billion to $26.0 billion [9, 10].

Abiotic stresses induced by climate change, including drought and heat, pose an increasing threat for all North American trees, including green ash [11]. In northeastern U.S. forests, greater precipitation and warmer temperatures have been recorded [12], and species ranges are expected to shift northward in response [13]. Climate change can also affect the spread of invasive pests [14, 15]. Changing climate may help the EAB to move further north into territory where winters are currently too cold to allow the larvae to survive [16], possibly more rapidly than the naturally slow range shifts expected of the trees themselves.

Molecular tools and genomic resources are less well established for most forest trees than for agricultural crops, although forestry applications show great promise [17, 18]. Green ash is one of the economically and ecologically important tree species facing devastating losses from pests and other environmental stresses for which genomic resources are needed to gain a greater understanding of molecular responses to stress. Transcriptome studies have been an effective means for identifying candidate genes utilized by trees to combat stress, in species such as cork oak (*Quercus suber*) [19], chestnut (*Castanea mollissima*) [20] and Douglas fir (*Pseudotsuga menziesii*) [21]. Transcriptome data also serve as a source of sequence-based genetic markers, enabling studies of population structure, genetic linkage mapping, quantitative trait loci (QTL) identification, and associations of phenotype with genotype. Such information provides powerful tools to advance pedigree-based breeding and selection programs as well as management of standing populations [17].

Currently, there is insufficient genomic information for green ash to undertake molecular-based tree improvement approaches. Little is known about ash response to stress at the gene level. A previously reported RNASeq resource pooling phloem tissue samples from green, white, black, blue and Manchurian ash species yielded over 58,000 assembled transcript sequences, a valuable resource for genetic marker design and initial functional characterization of genes expressed in ash phloem tissue, on which EAB feeds [22]. However, the sequence obtained in that study was all from healthy tissues pooled from all five species, making it impossible to assign sequences to individual species or to identify genes activated in response to stress. To expand the resources for green ash we conducted high-throughput RNA sequencing with a diversity of tissues and stress conditions, including cold, heat, drought, ozone, wounding, and EAB-feeding. The sequences were assembled into a reference transcriptome and differential gene expression between libraries was examined to identify general stress-response genes conserved across tissues and stress types.

## Methods

### Sampling and treatments

#### Parent tree treatments and tissue collection

Seed with wings, axial buds, terminal buds, leaflets, one-year-old twigs, xylem from three-year-old twigs and open pollinated seeds were collected from a healthy adult green ash tree located at the University of Missouri’s Agroforestry Research Center. From the same tree, attached leaflets were wounded by punching multiple holes with a paper punch across the leaf tissue, and one-year-old twigs were wounded by snapping the end of the twigs off. Tissues from the wound sites (broken twig end or leaf hole margin) were collected after 5 h and again after 24 h. DNA samples from this tree have been banked and are available upon request.

#### Seedling growth for stress treatments

Open pollinated seeds from the above parent tree were germinated in the greenhouse at the University of Missouri. At the age of 1 year, seedlings were shipped to Clemson University and to Pennsylvania State University for abiotic stress treatments. One-year-old open-pollinated seedlings were acclimated to the normal greenhouse environment for at least 1 month prior to abiotic stress treatments. Six biological replicates were used for all treatments levels for cold, heat, wounding, drought and ozone stress experiments on seedlings.

#### Seedling temperature stress treatments and tissue collection

Heat and cold treatments were conducted in a growth chamber with a cycle of cool fluorescent light followed by 8 h of dark. Heat-stressed tissues were collected after 24 h of exposure to 40 °C. Cold stress was induced by exposing 12 seedlings to 4 °C for 24 h. Tissues were collected from half of the seedlings immediately after cold stress (“cold stressed”). Tissues were collected from the other half of the seedlings 24 h after they were returned to the normal greenhouse environment (“cold stressed, recovery”).

#### Seedling drought stress treatments and tissue collection

Drought and mechanical wounding were performed in the greenhouse facility. For drought treatment, watering was withdrawn from two sets of seedlings: one set was for tissue collection, and the other set for petiole pre-dawn water potential measurements. When water potential in six out of seven surveyed seedlings dropped below −0.1Mpa, tissues were collected from six seedlings going through the same drought scheme as the surveyed ones.

#### Seedling mechanical wounding treatments and tissue collection

Mechanical wounding was introduced by punching four holes per leaf. Wounded leaves were then collected either 5 or 24 h after wounding. All tissues were collected in the morning, except for the 5 h after wounding time point, for which tissues were collected in the afternoon. Leaf, petiole, and root tissues were collected for heat, cold, and drought treatments; leaf and petiole tissues were collected for mechanical wounding as above.

#### Seedling ozone stress treatments and tissue collection

Greenhouse-acclimated seedlings (<10 parts per billion (ppb) ozone) were placed into Continuously Stirred Tank Reactor (CSTR) chambers. After at least 3 days of acclimation to the chambers, ozone was delivered as described by Heck et al. (1978) [23]. Ozone exposures were conducted for 28 days: <10 ppb ozone as control, 80 ppb, 125 ppb, and 225 ppb. Ozone was delivered in square-wave fashion, for 8 hr, 7 day/wk, with exposures beginning at 0900 h, ending at 1659, via a controllable micro metering system. Concentrations were monitored with a TECO Model 49 O_3_ analyzer and data logger/computer recording system. Leaf samples were collected at 3 time points (7 hr, 14 days, 28 days) after stress initiation with six biological replicates (seedlings) for each time point/treatment, for a total of 24 seedlings. After sampling on the 28th day, mechanical wounding was conducted in situ, with three leaves of each plant being wounded by multiple hole punches. Leaf samples from the hole margin were collected 24 h post-wounding (i.e. “29-day wounding”).

#### EAB larvae treatments

Tissues from four putatively EAB-resistant (PE19, PE21, PE22, and PE24) and two confirmed EAB-susceptible (PE36 and SUM) green ash genotypes were collected before and after exposure to EAB larvae. The EAB-feeding bioassay is described in [24]. Briefly, the two EAB-susceptible and four EAB-resistant genotypes were grafted and grown in the greenhouse for 2 years. Samples of bark and phloem of each genotype were acquired in the summer as untreated control tissues. Subsequently EAB eggs were placed under the bark around the stem of each graft for hatching. After 8 weeks of EAB larvae feeding, the insects were removed and the EAB-damaged phloem and bark tissues were sampled. Vouchers for the wild-collected green ash trees are available at USDA Forest Service, Northern Research Station, Project NRS-16 under voucher accessions FS-NRS16-241-2016 (tree PE36), FS-NRS16-242-2016 (tree PE19), FS-NRS16-243-2016 (tree PE21), FS-NRS16-244-2016 (tree PE22), and FS-NRS16-245-2016 (tree PE24). Tree PE36 was collected by Kathleen Knight and David Carey. Trees PE19, PE21, PE22 and PE24 were collected by Mary Mason and Dan Herms. Green ash tree SUM is the cultivar ‘Summit’ and was accessioned from Dawes Arboretum (D1991-0541). A voucher for this tree is also available at the USDA Forest Service, Northern Research Station, Project NRS-16 (FS-NRS16-240-2016).

### RNA extraction and library preparation

Tissue samples collected at the time points mentioned above were immediately flash frozen in liquid nitrogen and stored at −80 °C until RNA extraction. For stress experiments of seedlings with biological replicates, tissue samples for RNA extraction were pooled in equal amounts from each replicates per treatment per time point. Total RNA was isolated from ~1 g of frozen pooled tissue, using a modified CTAB method with lithium chloride precipitation [25]. RNA quality was assessed using an Agilent Bioanalyzer (Agilent technologies). cDNA libraries were prepared from the pooled RNAs for each treatment and time point for a total of 55 libraries. For each sample, 1ug of RNA was converted to cDNA using the Illumina TruSeq kit. The cDNA samples were sheared on a Covaris S2 to ~300 bp, following the manufacturer’s recommendation (Covaris, Woburn, MA). Size selection was performed on the Biomek FXp using the SPRIworks HT Reagent Kit. Each library was uniquely tagged with one of Illumina’s TruSeq LT DNA barcodes to allow library pooling for sequencing. Library quantitation was performed using Invitrogen’s Picogreen assay and the average library size was determined by running the libraries on a Bioanalyzer DNA 1000 chip (Agilent). Library concentration was validated by qPCR on a StepOne Plus realtime thermocycler (Applied Biosystems, Grand Island NY), using qPCR primers, standards and reagents from Kapa Biosystems (Wilmington, MA).

### Transcriptome sequencing and *de novo* assembly

Of the 55 RNASeq libraries for green ash, 41 were sequenced on both the Illumina MiSeq Desktop and the Illumina HiSeq 2000 sequencers (San Diego, CA), 12 were sequenced only on the MiSeq, and two were sequenced only using HiSeq 2000. For most libraries, quality was assessed by running the samples on an Illumina MiSeq sequencer and high throughput sequencing was carried out on an Illumina HiSeq 2000 sequencer at a read-length of 101 bp paired-end. All raw reads were deposited in the NCBI Short Read Archive (SRA) under the bioproject accession PRJNA273266. A summary of read statistics per library is provided in Additional file 1.

Raw sequences were trimmed using trimmomatic version 0.32 [26]. Trimmed reads generated from the MiSeq platform were assembled using Trinity pipeline version r20121005 [27]. Outputs from the Trinity assembly were further assembled with cd-hit version 4.6.1 to collapse isoforms [28]. The Trinity plugin TransDecoder was used to predict open reading frames (ORFs) in the assembly [29].

In supplemental files and public repositories, all transcript names begin with “Fraxinus_pennsylvanica_120313_” to indicate transcriptome origin and version. This part of the transcript name this has been removed from the text for brevity. For example, transcript “Fraxinus_pennsylvanica_120313_comp52211_c0_seq2” is referred to in the text as “comp52211_c0_seq2”.

### Assembly quality assessment

Three methods were used for assessing whether the assembly contains all or most of the green ash genes. First, CEGMA (Core Eukaryotic Genes Mapping Approach) version 2.5 was used to compare the assembled green ash transcriptome against the core set of eukaryotic genes [30]. Next, nine incremental assemblies were conducted with subsets of data to build a saturation curve and predict if new gene discovery would be likely with additional sequencing. The nine assemblies were performed with the same methodology previously described for the full assembly. Each additional assembly used all of the data from the previous assembly plus an additional set of tissues or treatments. The libraries included in each assembly and assembly statistics are provided in Additional file 2. A third quality assessment was performed by aligning all trimmed read pairs to the transcriptome with bowtie2 version 2.2.1 [31].

### Functional annotation and SSR discovery

Both the transcript sequences and the amino acid sequences from the predicted ORFs were queried against the Swiss-Prot protein database and the plant taxonomic division of the TrEMBL protein database [32] using BLAST+ version 2.2.22 [33]. Amino acid sequences were subjected to InterProScan version 5.4–47.0 searches to predict protein family membership and identify conserved domains [34]. Gene ontology (GO) terms [35] were assigned using the InterProScan software [36].

Simple sequence repeats (SSRs) were identified from transcripts. Di-, tri-, and tetra- nucleotide repeats were only reported if they met the following criteria: di-nucleotide repeats with 8–200 copies, tri-nucleotide repeats with 7–133 copies, and tetra-nucleotide repeats with 6–100 copies. SSRs were flagged as compound if they were adjacent or separated by less than 15 bases. Primer3 v2.3.6 was used to design primers flanking the SSRs, excluding all compound SSRs. Sequences were masked for low complexity regions with dustmasker [37] prior to primer design. The following parameters were altered from the default: primer_product_size_range = 100–450, primer_min_tm = 55.0, primer_max_tm = 65.0, primer_min_gc = 40, primer_max_gc = 60, primer_max_poly_x = 3, primer_gc_clamp = 2. The perl script used to extract these sequences and run Primer3 is available at via GitHub [38]. The spreadsheet output by this script is available as Additional file 3 and contains summary statistics, SSR locations and primer sequences.

### Gene expression across tissues and treatments

HTSeq version 0.6.1p1 was used to produce raw read counts for each transcript per library [39]. To account for variations among gene length and library size, these counts were converted to the metric RPKM (reads per kilobase per million mapped reads) [40]. To calculate the number of transcripts expressed in each sample, a minimum expression cut-off of RPKM > 0.1 was used. As previously described [41–43], log2(RPKM +1) normalized values were used for clustering; adding one was necessary to prevent a log2 transformation from calculating undefined values in cases of zero values. Pearson correlations were calculated using the result of the log2-transformations. A distance matrix was constructed using these values. Hierarchical clustering was performed using the hclust function with average distance.

### Differential expression analysis

The R package DESeq2 version 1.63 was used to determine statistically significant differentially expression [44]. Raw counts from HTSeq were provided as input. All comparisons used the default Wald test except the Ozone libraries where the likelihood ratio test (LRT) was utilized. Principal component analyses (PCA) were also calculated with the DESeq2 package. The R code utilized to generate the results is available at GitHub [45] and the lists of differentially expressed putative unique transcripts (PUTs) are available in Additional file 4.

The set of up and down differentially expressed PUTs were each assessed for GO term enrichment using the Cytoscape application BiNGO v3.03 [46], an often used tool for assessing GO enrichment in transcriptome studies [47–49]. The R scripts and raw data files used to generate figures, including the cluster analysis and GO term enrichment in BiNGO, are archived publicly at https://github.com/statonlab/green_ash_rnaseq. All GO enrichment results are listed in Additional file 5.

## Results and discussion

### Transcriptome sequencing and *de novo* assembly

Transcriptome sequencing of 55 green ash RNA samples spanning a variety of tissues and treatments yielded over 99 Gb of sequence data. Sample libraries encompass EAB damage, specialized tissues, mechanical wounding, heat exposure, drought exposure, cold exposure, ozone exposure, and ozone exposure plus mechanical wounding (Table 1). A stringent filtering and *de novo* assembly pipeline was used to produce 107,611 PUTs. The PUTs have an average length of 818 nucleotide base pairs and an N50 of 1327 bases. A total of 52,899 open reading frames (ORFs) could be identified from 47,069 PUTs (43 %). PUTs with more than one ORF may represent operons from plastid or mitochondrial genomes, chimeras, or misassemblies where a 1–2 base insertion/deletion shifted the ORF. PUTs without an ORF were of considerably shorter length: 409 bases in average length of PUTs without ORFs vs. 1342 bases average length of PUTs with ORFs. This may indicate that lack of a captured start or stop codon impeded ORF identification or that the reads originated from noncoding RNAs.

**Table 1:** Samples for sequencing

Tissue	Source	# Reads
Leaves, ambient ozone for 7 h	6 seedlings, pooled	419,064
Leaves, 80 ppb ozone for 7 h	6 seedlings, pooled	457,118
Leaves, 125 ppb ozone for 7 h	6 seedlings, pooled	495,728
Leaves, 225 ppb ozone for 7 h	6 seedlings, pooled	500,118
Leaves, ambient ozone for 14 days	6 seedlings, pooled	450,932
Leaves, 80 ppb ozone for 14 days	6 seedlings, pooled	427,298
Leaves, 125 ppb ozone for 14 days	6 seedlings, pooled	507,634
Leaves, 225 ppb ozone for 14 days	6 seedlings, pooled	483,742
Leaves, ambient ozone for 28 days	6 seedlings, pooled	440,864
Leaves, 80 ppb ozone for 28 days	6 seedlings, pooled	486,950
Leaves, 125 ppb ozone for 28 days	6 seedlings, pooled	516,644
Leaves, 225 ppb ozone for 28 days	6 seedlings, pooled	318,888
Leaves, 80 ppb ozone for 28 days, wounding after 28th day, 29 days total	6 seedlings, pooled	13,160,726
Leaves, 125 ppb ozone for 28 days, wounding after 28th day, 29 days total	6 seedlings, pooled	11,583,960
Leaves, 225 ppb ozone for 28 days, wounding after 28th day, 29 days total	6 seedlings, pooled	12,506,320
Leaves, ambient ozone for 28 days, wounding after 28th day, 29 days total	6 seedlings, pooled	10,723,914
Unstressed leaves	6 seedlings, pooled	24,953,504
Unstressed petioles	6 seedlings, pooled	30,230,264
Unstressed roots	6 seedlings, pooled	29,378,320
Wounded leaves 5 h	6 seedlings, pooled	24,619,536
Wounded leaves 24 h	6 seedlings, pooled	27,899,560
Wounded petioles 5 h	6 seedlings, pooled	23,312,492
Wounded petioles 24 h	6 seedlings, pooled	25,669,498
bark and phloem after EAB feeding	Tree 19	23,628,074
bark and phloem control	Tree 19	52,011,186
bark and phloem after EAB feeding	Tree 21	27,585,376
bark and phloem control	Tree 21	25,016,884
bark and phloem after EAB feeding	Tree 22	21,570,304
bark and phloem control	Tree 22	29,090,224
bark and phloem after EAB feeding	Tree 24	26,814,566
bark and phloem control	Tree 24	26,043,308
bark and phloem after EAB feeding	Tree 36	26,342,646
bark and phloem control	Tree 36	26,495,904
bark and phloem after EAB feeding	Tree Summit	32,053,344
bark and phloem control	Tree Summit	59,097,222
Cold stressed leaves (4C for 24 hr, recovery for 24 hr)	6 seedlings, pooled	29,361,894
Cold stressed petioles (4C for 24 hr, recovery for 24 hr)	6 seedlings, pooled	15,174,806
Cold stressed roots (4C for 24 hr, recovery for 24 hr)	6 seedlings, pooled	21,691,182
Cold stressed leaves (4C for 24 hr)	6 seedlings, pooled	15,625,080
Cold stressed petioles (4C for 24 hr)	6 seedlings, pooled	18,679,338
Cold stressedroots (4C for 24 hr)	6 seedlings, pooled	18,870,828
Drought stressed leaves (<1.0 Mpa)	6 seedlings, pooled	15,984,282
Drought stressed petioles (<1.0 Mpa)	6 seedlings, pooled	20,242,284
Drought stressed roots (<1.0 Mpa)	6 seedlings, pooled	18,864,744
Heat stressed leaves (40C for 24Hr)	6 seedlings, pooled	14,167,240
Heat stressed petioles (40C for 24Hr)	6 seedlings, pooled	13,049,768
Heat stressed roots (40C for 24Hr)	6 seedlings, pooled	13,415,506
Unstressed leaves (control for wounded)	Adult tree	10,829,406
Wounded leaves	Adult tree	14,083,066
Unstressed 1 year old twigs	Adult tree	11,524,160
Wounded 1 year old twigs	Adult tree	14,157,548
3-year-old xylem	Adult tree	8,191,208
Seed wings	Adult tree	36,521,672
Axial buds	Adult tree	13,411,952
Terminal buds	Adult tree	14,531,836

### Assembly completeness

Three strategies were employed to test the completeness of the transcriptome: comparison to CEGMA (Core Eukaryotic Genes Mapping Approach), alignment of reads to the transcriptome, and saturation analysis. CEGMA includes a database containing 248 highly conserved eukaryotic genes and a computational method for assessing the presence of these genes in a dataset [30]. Comparison of the green ash transcriptome to the CEGMA dataset indicates that all core eukaryotic genes are present in the final assembly. The majority, 238 genes or 96 %, were found to be complete in length in the green ash transcriptome. For the remaining ten genes, green ash transcripts were found but span only a portion of the expected gene length.

**Fig. 1:**
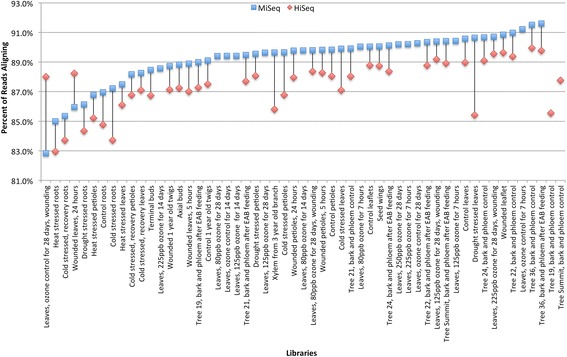
Read alignments by sequencing platform. The percent of reads that successfully aligned to the final transcriptome for each sequencing run ranged from 83 to 92 %. Reads produced from the Illumina MiSeq (*blue squares*) were slightly more likely to align than reads from the Illumina HiSeq (*red diamonds*). If the same library was run on both platforms, then the two experiments are linked by a line

To assess how well the final assembly represented all of the sequenced reads, the reads were aligned to the PUTs. A slight difference in rates of alignment for libraries from two different sequencing instruments was detected. For reads from the MiSeq instrument, an average of 89 % of reads aligned with a range for the 53 individual libraries from 83 to 91 %. Reads from the HiSeq aligned on average at a rate of 87 %, with a range of 83 to 90 % for individual libraries. Less than 3 % of all read pairs aligned discordantly, i.e. two paired reads aligned to different transcripts. For all but two of the libraries sequenced on both platforms, the MiSeq reads aligned at a slightly higher rate, about 1.6 % more often, than the reads from the HiSeq for the same library (Fig. 1). However, for individual libraries sequenced on both platforms, the rate of alignment from the MiSeq is correlated to the rate for the HiSeq (*R*^2^ = 0.68), confirming that library quality can be assessed effectively on a MiSeq platform prior to higher-throughput sequencing on a HiSeq.

**Fig. 2:**
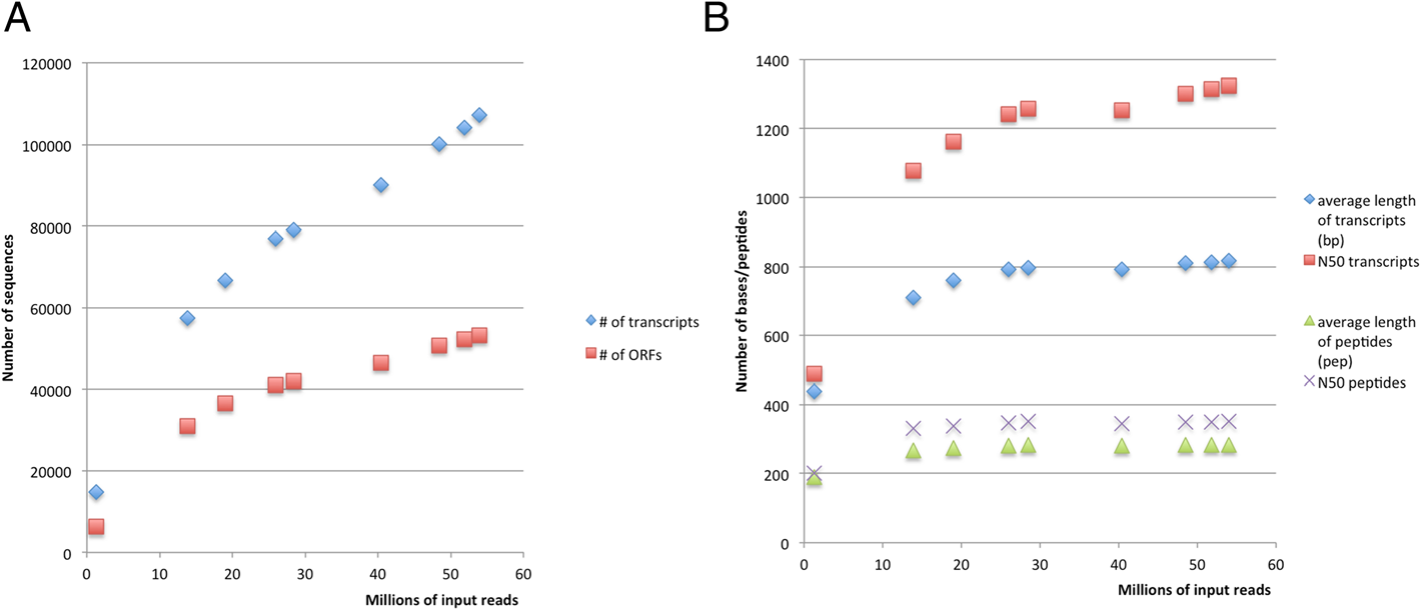
Gene discovery saturation curve. A step-up method of assembly was completed in order to determine if additional sequencing is likely to yield new transcripts. For increasing numbers of input reads, the total number of transcripts and proteins continued to increase (**a**). For the average length and N50 of the transcripts, additional reads induced small increases for transcripts while predicted ORFs were unchanging after 30 million input reads (**b**)

Saturation analysis was carried out to detect the incremental new gene discovery from the addition of new RNA libraries. A rarefaction curve was generated by producing nine assemblies using subsets of data. The smallest data set assembled was a single RNA library, with additional libraries added to each subsequent assembly until all libraries were included. The incremental assembly size ranged from 14,723 transcripts to 107,363 transcripts depending on total data included in the assembly; more sequencing libraries resulted in more transcripts with each addition of data (Additional file 2). While the overall number of transcripts and ORFs are increasing with the addition of data, the total number of identified ORFs increased much more slowly, indicating that additional sequencing would likely yield few new ORFs (Fig. 2a). From an assembly with input data of 51.8 M reads to 54.0 M reads, 3149 new transcripts were found but only 1053 new ORFs were discovered. Regarding the length of the transcripts, both N50 and average length are increasing only slightly at the largest data input sizes (Fig. 2b): one additional base pair in N50 length per addition of 200,000 input reads and one additional base pair in average transcript length per addition of 500,000 input reads. Saturation was reached for peptide length for both N50 as well as average length. From the fourth largest assembly with an input of 26.0 M reads to the final assembly of 54.0 M reads, the average and N50 lengths increased by only four and five amino acids, respectively.

The three quality assessment metrics indicate that the green ash de novo transcriptome represents the majority of expressed genes based on presence of conserved eukaryotic genes, the alignment of the majority of reads back to the assembly, and a saturation analysis indicating that few new ORFs are likely to be discovered with additional sequencing. The strategy of sequencing RNA from a variety of green ash tissues and treatments effectively maximized the sampling of all genes, yielding a rich transcriptome sequence resource for further genomic and genetic work in ash.

### Functional annotation and SSR discovery

To provide functional annotation for the green ash PUTs, a combination of sequence similarity, protein domain searches and GO term assignments were conducted. BLAST searches [50] against the Swiss-Prot and plant TrEMBL databases [32] were conducted to compare the green ash PUTs to previously sequenced and annotated proteins. For transcript sequences, 46 % matched at least one Swiss-Prot accession and 63 % matched at least one plant protein from TrEMBL. The inferred homology results support the ORF-predictions; 98 % of PUTs with an ORF matched a known protein while only 36 % of PUTs without a predicted ORF matched a known protein. PUTs without a match to known proteins may be non-coding RNAs, genes that have significantly diverged from available reference sequences, or erroneous sequence data. The predicted protein sequences from the ORFs were characterized for homology to protein families and domains by InterProScan [36] (Additional file 7), yielding additional functional information for 45,893 protein sequences (87 %). InterProScan also assigned Gene Ontology (GO) terms; 29,666 proteins (56 %) were assigned at least one GO term. The GO terms indicate that a variety of different genes were captured, with 679 biological process, 914 molecular function and 227 cellular component GO terms assigned.

Extraction of SSRs yielded a total of 1956 individual SSRs and 5 compound SSRs from 1937 transcript sequences. SSRs were relatively rare, with less than 2 % of transcripts yielding an SSR and an average of one SSR per 44.8 kilobase (kbp) of transcript. Excluding compound SSRs, di-nucleotides were the most common making up 87.7 % of the total. The second most common was tri-nucleotides at 11.9 %. Tetra-nucleotides make up less than 1 % of the total. Primers were successfully designed to flank 486 of the repeats (Additional file 3). Repeats with primers were cataloged for 431 di-nucleotide SSRs with a range of eight to 11 motif copies, 54 tri-nucleotide repeats with a range of seven to 12 motif copies, and a single tetra-nucleotide with six motif copies.

### Expression across tissues and treatments

The high depth of reads obtained during RNA sequencing enables comparison of transcriptome expression patterns across different tissues and treatments. Libraries with fewer than 1 million sequenced reads were excluded from this analysis due to possibly insufficient depth to capture rarely expressed transcripts, leaving 43 libraries for analysis. Individual RNA samples were found to express from 76,861 to 99,706 PUTs with two libraries found as outliers with significantly lower counts: 3-year-old xylem tissue with 52,419 expressed PUTs and EAB fed bark and phloem from Tree 19 with 48,605 expressed PUTs. Fewer identified transcripts may be indicative of library preparation variation or an actual lower number of genes expressed biologically.

**Fig. 3:**
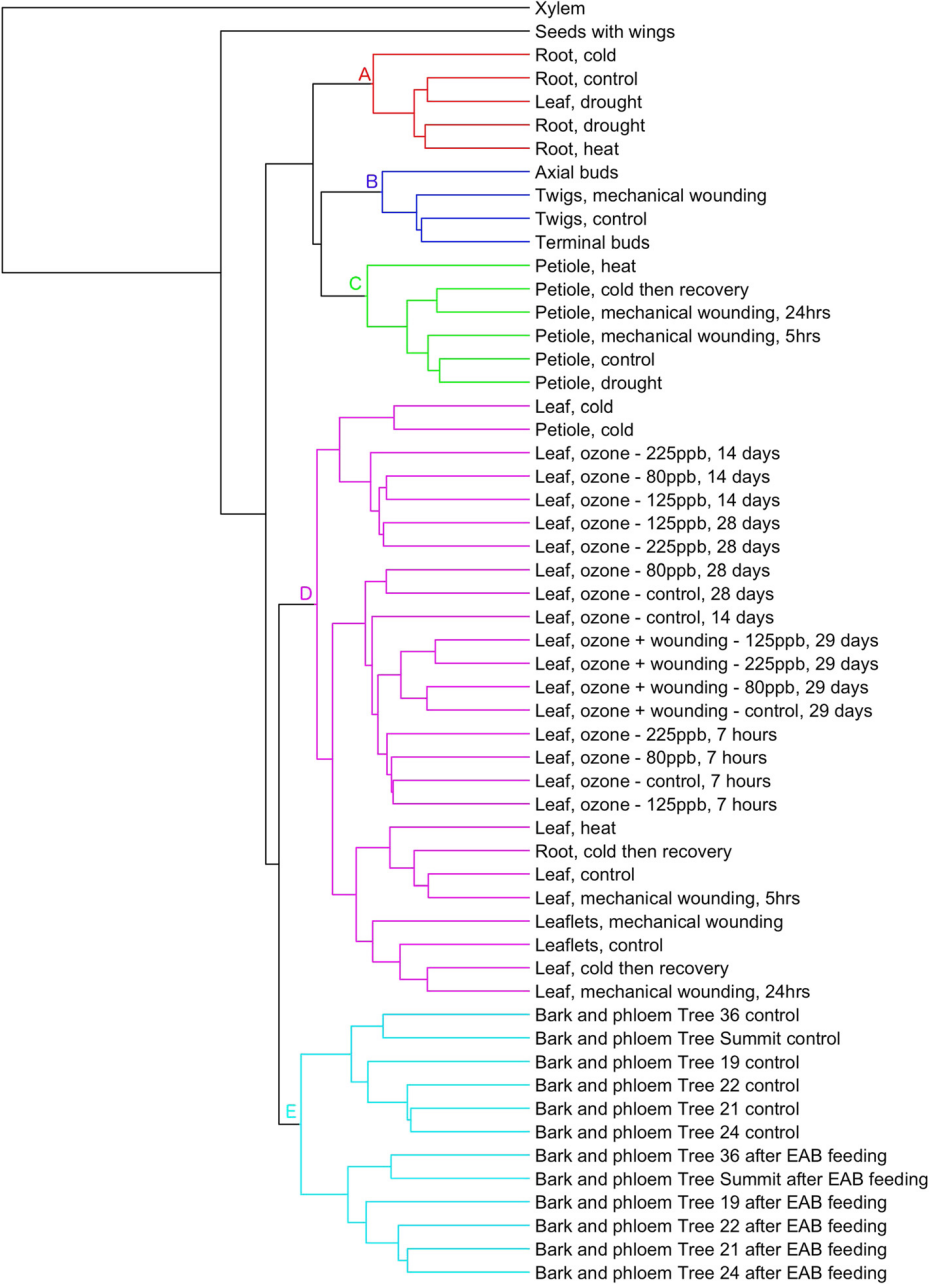
Hierarchical clustering of tissues. The 55 *green* ash RNA samples were clustered by normalized *read* counts across all PUTs. Clusters (**a**–**e**) highlight groups of samples originating from similar tissues and/or experimental treatments

Hierarchical clustering of all libraries was performed to determine which samples had similar expression patterns (Fig. 3). The tissue type was found to be the strongest common element for clustering, indicating that tissue-specific transcription patterns, even under stress, are conserved. Cluster A includes mostly leaf tissue samples, including control, wounding, cold and ozone plus wounding conditions. Cluster B includes all of the petiole tissue samples and the heat stressed leaf sample. Cluster C includes the majority of the bark and phloem tissues; the exceptions include Tree 19 samples as well as the Tree Summit control sample. Cluster D includes primarily root samples, and cluster E includes twig and bud tissue samples exclusively. Interestingly, twigs and buds from a mature green ash did not group with the bark and phloem samples from two-year-old grafts. Cluster F includes the majority of ozone stressed leaf tissue samples with a single exception, the ozone stressed leaf tissue samples with wounding on the 28th day. They did not group with other leaves, but this may be due to a batch effect; all samples in cluster F have only MiSeq data, resulting in overall lower depth than all other samples sequenced.

### Differential expression analysis

The RNA sequencing of both control and stressed green ash tissues enables an initial inquiry into the regulation of transcripts under each of six types of stress: EAB feeding, cold, drought, heat, mechanical wounding and ozone (Table 2). Statistically significant changes of either increased or decreased transcript abundance between stressed tissues and control conditions were determined with a cutoff of *p* < 0.05 using the package DESeq2 for each type of stress. For the EAB test, the six genotypes were sequenced invidually and used as biological replicates. For the remaining stress tests, six biological replicates were produced in the greenhouse, and the RNA from each replicate was pooled prior to sequencing.

**Table 2:** Differential expression and GO term enrichment results

	Transcripts increasing in expression			Transcripts decreasing in expression		
Test	# transcripts	# Enriched GO plant slim terms	# transcripts with putative function	# transcripts	# Enriched GO plant slim terms	# transcripts with putative function
EAB - Control tissues vs Infested Tissues	6391	12	5346	6884	10	5534
EAB - Susceptible vs Resistant, Pre-EAB Feeding	750	5	460	899	2	497
EAB - Susceptible vs Resistant, Post-EAB Feeding	545	1	336	580	2	351
Cold-stressed tissues vs control tissues	3196	9	2336	456	0	342
Drought-stressed tissues vs control tissues	13	2	12	82	7	66
Heat-stressed tissues vs control tissues	502	7	386	1114	8	984
Mechanically-wounded tissues after 5 h vs control tissues	237	5	208	252	3	217
Mechanically-wounded tissues after 24 h vs control tissues	307	1	244	653	3	544
Tissues at 4 levels of ozone across 3 time points^a^	350^a^	15^a^	342^a^	^a^		

Statistical tests were conducted for each stress condition to determine genes with increased or decreased expression (adjusted *p*-value < 0.01). These genes were assessed for shared biological processes or molecular functions via ontology enrichment based on the subset of GO plant slim terms

^a^Transcripts responsive to ozone. A likelihood ratio test was used to identify any transcripts responsive to ozone treatment across multiple time points, allowing PUTs with more complex patterns, for example initially up regulated, then down regulated, to be included

EAB is a primary threat to green ash trees. To understand defense responses on a molecular level, six genotypes of ash were assessed at two time points: pre-EAB feeding and 8-weeks post-EAB larval hatch and feeding. A Wald test of the data found 13,275 differentially expressed PUTs, 6884 of which had lower expression after feeding, and 6391 of which had higher expression after feeding. This large difference includes the response to EAB feeding but also includes seasonal changes and other environmental factors that impacted the seedlings during the 8 weeks of feeding.

**Fig. 4:**
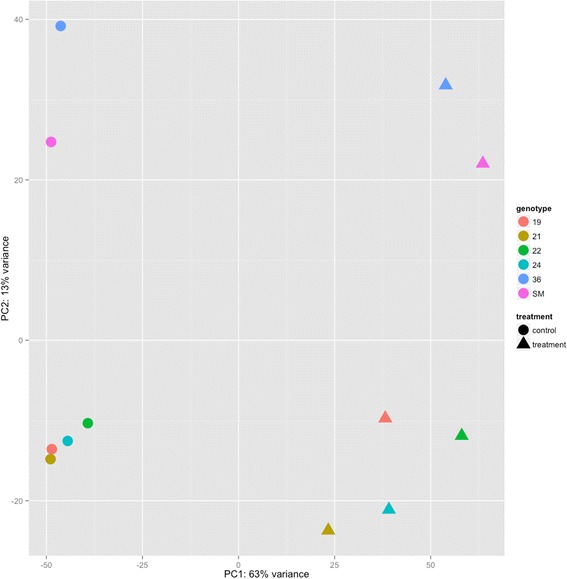
Principal component analysis (PCA) of *green* ash EAB feeding experiment. A plot showing the results of a principal components analysis of *Green* ash EAB feeding differential expression results. The x-axis plots the variance of the first principal component and the y-axis plots the variance of the second principal component

The genotypes utilized for the EAB feeding bioassay were selected for their differing response to EAB; four were identified as putatively resistant to EAB. These ‘lingering ash’ trees were found to have a healthy canopy after EAB had caused over 95 % mortality of surrounding ash trees. The remaining two genotypes are both known to be susceptible to EAB. For statistical analysis, the resistant genotypes represented four biological replicates and the susceptible genotypes were used as two biological replicates. This allows for an additional comparison of interest, i.e. the difference in response by four EAB-resistant green ash genotypes in comparison to two EAB-susceptible genotypes. A principal components analysis (PCA) of the PUT expression patterns among the twelve samples suggests that susceptibility has a detectable association with expression; 63 % of variance across samples corresponds to treatment. A secondary variance component of 13 % separates resistant versus susceptible genotypes (Fig. 4).

We performed a test for expression differences between resistant and susceptible genotypes before larval feeding to determine if the tree’s transcriptome patterns are different prior to insect exposure. For pre-feeding samples, 899 PUTs are down-regulated and 742 PUTs are up-regulated in the resistant genotypes relative to the susceptible. We also tested for differences in resistant and susceptible genotypes post-feeding to identify active plant defense response patterns. In post-feeding samples, 580 PUTs are less expressed and 545 PUTs are more expressed in resistant genotypes. The candidate PUTs identified in this experiment may prove useful for further studies regarding the molecular mechanisms of natural resistance to EAB.

Plant response to insect herbivory is complex and may be induced by detection of nonself compounds as well as by signals sent from damaged cells [51]. Tissue damage may also be caused wind, hail, and other mechanical factors. To explore ash response specifically to wounding, experiments were conducted on leaves, petioles and twigs. Experimentation included single biological replicates of tissues (leaf, twig) on an adult tree 24 h after damage and six biological replicates of seedling tissues (petiole, leaf) 5 and 24 h after being damaged. Using all tissues types in the Wald statistical test, we identified genes with significantly different abundances correlated to mechanical wounding after 5 h and after 24 h. For 5 h post mechanical wounding, we found 237 up-regulated PUTs and 252 down-regulated PUTs. Additional differentially expressed PUTs were found after 24 h: 307 up-regulated and 653 down-regulated. Some genes were identified as differentially expressed at both time points: 109 genes were down regulated at both time ponits and 16 were up regulated at both time points.

#### Climate change stressors

The alterations of Earth’s climate will impose increased abiotic stresses with implications for the adaptation and survival of ash species. We conducted analyses of four stressors expected to increase in severity in native forests as part of climate change: heat, cold, drought, and ozone (which also serves as a general oxidative stress for which accurate dose-response investigations can be conducted under controlled conditions). Experiments were conducted with six biological replicates per condition: control, heat, cold and drought conditions across leaf, petiole, and root tissues of green ash seedlings. For all statistical tests of differential gene expression, the three tissues were considered together to provide increased statistical power and to discover transcripts implicated in whole plant response to stress. Drought produced significantly fewer differentially expressed PUTs than the other stressors, with 13 PUTS increasing in expression and 82 PUTs decreasing. Heat stress induced up-regulation for 502 PUTs and down-regulation for 1114 PUTs. Cold stress induced the most changes of the three stress types, with 3196 increasing PUTs and 456 decreasing. An additional oxidative stress, increasing ozone, was assayed only for leaves, but sampled across three time points (7 h, 14 days, 28 days) and four ozone concentrations (atmosphere, 80 ppb, 125 ppb, 225 ppb). We used a likelihood ratio test (LRT) to identify 350 PUTs that responded to changes in ozone levels. Four experimental treatments involved a combination of mechanical wounding and ozone stress; neither a Wald test nor a LRT yielded statistically significant gene associations for these samples versus control tissues. This is surprising given that each treatment independently showed differential gene expression. Possibly, crosstalk between ozone stress and mechanical wounding differs with different ozone levels, and the differences in gene expression response in each library led to a lack of statistical power in detecting additional differentially expressed genes after wounding.

#### GO term enrichment across stress responses

**Fig. 5:**
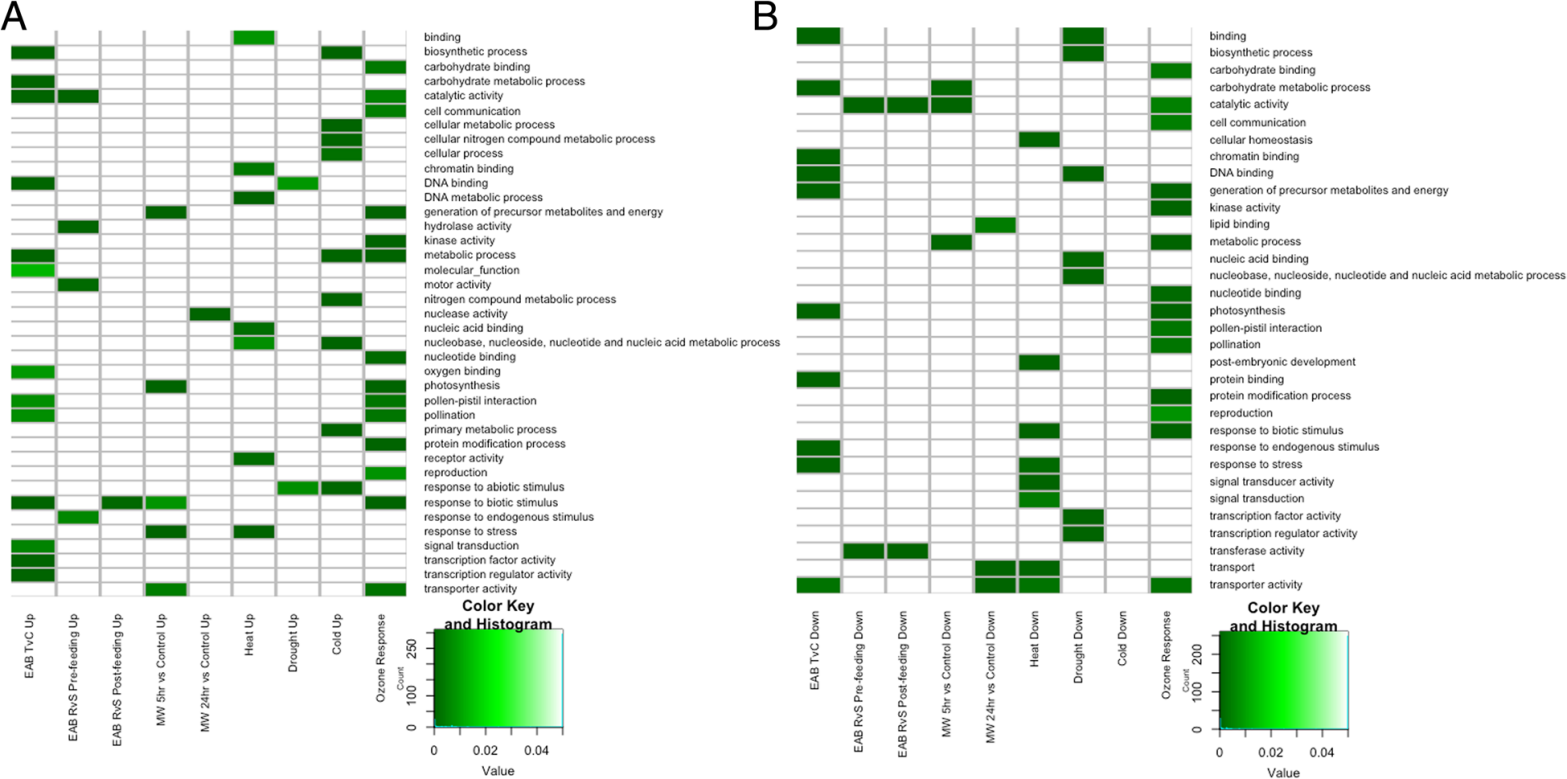
GO slim term enrichment heatmap across abiotic stresses. An enrichment analysis was used to identify significant GO terms across the list of increased (**a**) and decreased (**b**) PUTs for each stress condition. A *p*-value cut-off of 0.05 indicates a GO term is significantly more prevalent in the list than would be expected by chance. *Darker green* indicates a smaller *p*-value for the GO term association, and values above the cutoff *p*-value (0.05) are *white*

For each list of differentially expressed PUTs from different stress types, GO term enrichment was performed in order to identify molecular pathways and processes involved in stress response in green ash (Fig. 5). Expected functions were found in many experiments, such as genes in the category “response to abiotic stimulus” during drought and cold and “increased response to stress” under mechanical wounding and heat stress conditions. Many GO terms were identified in more than one stress condition, indicating overlap in the stress response pathways despite different stimuli. Unfortunately, many of the PUTs could not be functionally annotated and thus were not included in the GO term enrichment analysis. More information about these PUTs or their homologs in other plants is likely to illuminate additional pathways and biological processes of interest in response to environmental stresses relevant to climate change.

#### Genes shared across multiple types of stress

The detection of basal level stress response genes provides an opportunity to identify genes responding to multiple independent stressors. For most experiments, the biological samples were pooled at the sequencing level, reducing statistical power to detect underlying variability. However, the genes consistently detected as differentially regulated across multiple stresses are good candidates for further research in green ash defense response. No PUTs were found to be consistently up or down-regulated across all six stress types (ozone, drought, heat, cold, mechanical wounding, and EAB feeding). Two PUTs were down-regulated across 4 stresses, and although both had sequence similarity to plant genes in the protein database, their functions are as of yet uncharacterized. Seventy-six PUTs were up-regulated and fifty-seven transcripts were down-regulated across different combinations of three different stress treatments (Table 3).

**Table 3:** Transcripts with significantly different expression across multiple types of stress

Stressors	Increased or decreased expression	Total overlaps
Heat/Cold/EAB	Increased	11
Heat/Cold/Ozone	Increased	1
Heat/MW5hr/Ozone	Increased	1
Cold/MW5hr/EAB	Increased	3
Cold/EAB/Ozone	Increased	54
MW5hr/MW24hr/EAB	Increased	1
MW24hr/EAB/Ozone	Increased	1
Heat/Drought/Cold	Decreased	4
Heat/Drought/EAB	Decreased	5
Heat/Cold/EAB	Decreased	19
Heat/Cold/Ozone	Decreased	4
Heat/MW5hr/Ozone	Decreased	1
Heat/MW24hr/EAB	Decreased	2
Heat/MW24hr/Ozone	Decreased	1
Heat/EA/Ozone	Decreased	7
Drought/Cold/EAB	Decreased	1
Cold/EAB/Ozone	Decreased	2
MW5hr/MW24hr/EAB	Decreased	5
MW5hr/MW24hr/Ozone	Decreased	6
Heat/Drought/Cold/EAB*	Decreased	1
Heat/Cold/EAB/Ozone*	Decreased	1

For two genes, a decrease in expression under stress was found in four of the seven experiments (*). A set of 151 PUTs were identified as significantly differentially expressed in three of the seven experiments; 72 were more expressed under stress while 59 were less expressed under stress

**Fig. 6:**
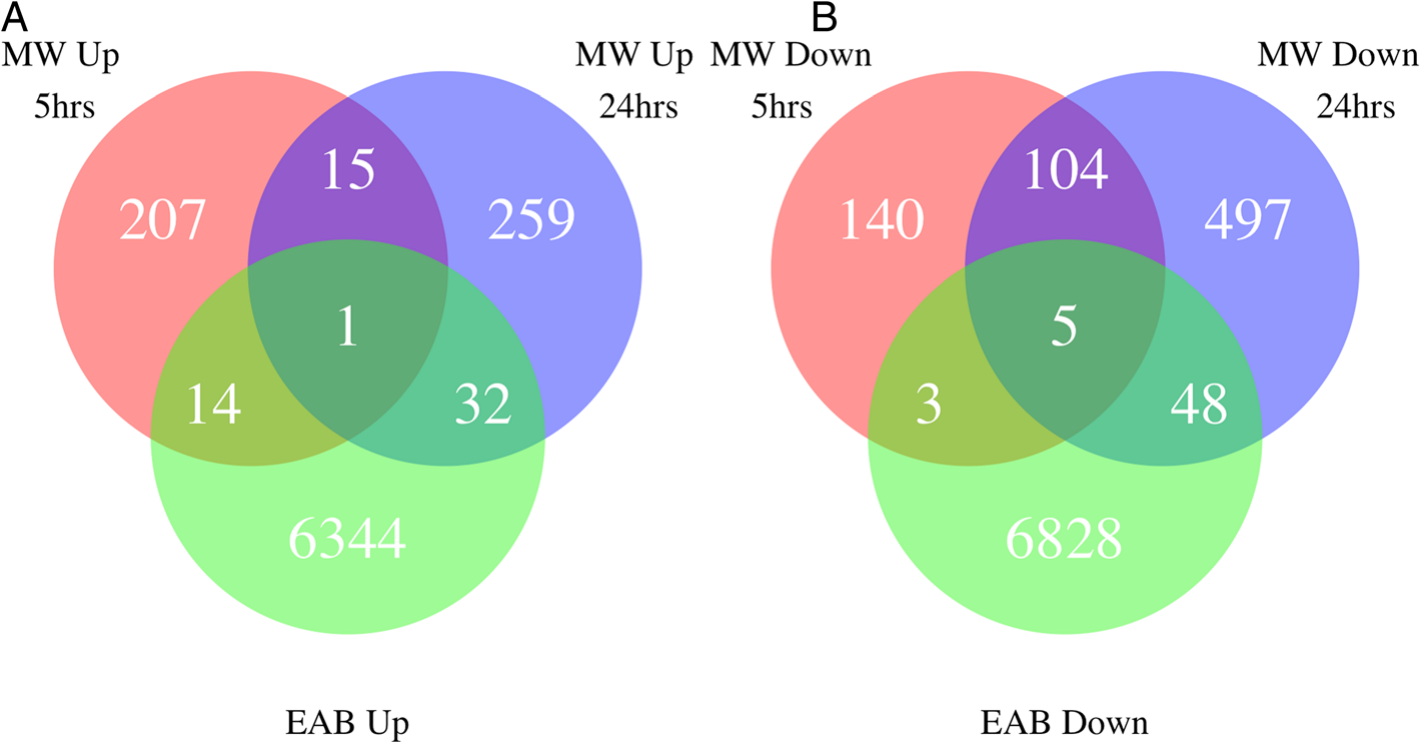
EAB and mechanical wounding venn diagram. Venn diagrams of increased (**a**) and decreased (**b**) differentially expressed genes shared among three experiments: emerald ash borer damage, 5 h after mechanical wounding, and a day after mechanical wounding

Mechanical wounding is one component of the stress caused by insect feeding. We identifed six PUTs with statistically significant changes in both wounding experiments and in the EAB response (Fig. 6). One transcript, comp54917_c0_seq1, had increased expression across both stresses and five transcripts had consistently decreased expression (comp52211_c0_seq2, comp59636_c0_seq1, comp27672_c0_seq1, comp47794_c0_seq1, and comp64971_c1_seq10). No functional annotation information was found for the up-regulated transcript or for two of the down-regulated transcripts. The remaining three down-regulated transcripts are an NRT1/PTR and two beta-glucosidases. The NRT1/PTR family of genes has nitrate transmembrane transport activity and is involved in auxin transport, a known phytohormone signaling system induced by biotic stress. Comp47794_c0_seq1 and comp64971_c1_seq10 are both beta-glucosidase genes, which have involvement in herbivory response as well as other forms of stress response [52, 53]. These results suggest that these genes may be important in response specifically to tissue damage.

Among three of the climate change stress agents (cold, heat, drought), three PUTs were found to be increased, two of which have homology to the REVEILLE gene, known for its involvement in circadian rhythm and as a negative regulator of cold tolerance [54]. The third up-regulated PUT is an ascorbate-specific transmembrane electron transporter, also referred to as cytochrome b561, which is critical to ascorbate recycling. Ascorbate synthesis and signaling have a known role in oxidative stress tolerance and are induced by multiple types of abiotic stress [55]. In a different combination of climate change stressors (heat, cold, ozone), a shared up-regulated PUT is germacrene-D synthase. Germacrene-D is a sesquiterpene, which are known to act as mediators in plant-environment interactions. Sesquiterpenes are known to be involved in herbivore defense in trees [56, 57], although they have been implicated in plant abiotic stress response as well [58]. For the same climate change stresses (heat, cold, ozone), four PUTs are commonly down-regulated. One down-regulated PUT resembles Arabidopsis gene YLS9, which is involved in the innate immune system and is induced by biotic stresses such as viruses [59].

The remaining PUTs with shared expression patterns across three conditions spanned both biotic and abiotic stresses. More differentially expressed transcripts were shared among cold, EAB feeding, and ozone treatments than any other combination of stresses, with 54 up-regulated and two down-regulated transcripts in common. Many of the genes regulated in common have previously been identified in stress response in other plants. Transcription factors are critical to inducing downstream transcriptional changes, and in green ash trees we identified three PUTS with strong homology to Arabidopsis WRKY transcription factor family members 30, 31, and 33: comp52191_c0_seq1, comp64498_c0_seq7, and comp60267_c0_seq1, respectively. WRKY’s have diverse biological functions but are particularly associated with response to biotic and abiotic stresses; they are thought to contribute to early signaling to activate adaptive responses [60]. Another shared PUT that is likely to be involved in early signaling (comp57076_c0_seq1) is a mitogen-activated protein kinase (MAPK), which have been characterized across a number of plant species to express immediately upon abiotic or biotic stress to induce immune responses [61]. Two scarecrow-like (SCL) transcription factors were found to be up-regulated in cold, mechanical wounding and EAB exposure. SCLs have been previously identified in response to salt and drought [62], particularly in root tissues [63].

Downstream of initial transcription factor and signaling cascades, phytohormones are well characterized signaling mechanisms that mediate plant defense. In the green ash stress libraries, we identified both up and down-regulated PUTs with associations to phytohormone signaling. Two up-regulated PUTs, comp68432_c0_seq1 and comp52044_c0_seq1, resemble AZF2 and ZAT10 in Arabidopsis respectively. Both of these genes are implicated in jasmonate (JA) signaling [64] and act to inhibit plant growth under abiotic stress conditions including drought and cold [65–67]. Another transcription regulator, ZAT11 (comp51423_c0_seq2) was also increased in response to multiple types of stressors and functions to repress transcription during stress, for example in metal exposure [68], drought, cold and high salinity [65]. A down-regulated PUT, comp54600_c0_seq1, is homologous to the transcription factor bHLH14 that acts to negatively regulate JA responses, and its down-regulation is important for effective JA-mediated plant defense response for biotic stress [69]. Other phytohormones are also likely involved in green ash defense; down-regulated comp61040_c0_seq5 resembles a Gibberellin (GA) 20 oxidase, which functions in the formation of bioactive GA. GA is known to function in pathogen defense, and in the case of this particular gene, knockouts in rice demonstrated increased transcription of defense genes and improved resistance to pathogens [70, 71].

Accumulation of harmful, cell-damaging reactive oxygen species (ROS) may be generated by both biotic and abiotic stresses. In the course of defense response, ROS molecules can also act as a signaling mechanism for stress tolerance. Thus ROS levels must be tightly controlled by molecular mechanisms to balance these two roles. PUTs involved in ROS were detected in the green ash defense responses. PUT comp45637_c0_seq1, up-regulated in defense response in green ash, is a metallothionein-like protein, which can scavenge ROS molecules. Metallothionein type 2 proteins have been associated with response to heat stress in rice [72], oxidative stress in cork oak [73], and heat and drought stress in the halophyte *Salicornia brachiata* [74]. A reticuline oxidase-like protein, comp53767_c0_seq1, is also up-regulated; the most closely related homolog in Arabidopsis is up-regulated in response to oxidative stress as well [75]. Other up-regulated PUTs (comp62432_c0_seq4, comp50698_c0_seq3) have identity to an oxidoreductase and a stellacyanin, both involved in cellular redox reactions.

## Conclusions

The reference transcriptome generated for green ash, with extensive functional annotation and annotated SSRs, is a valuable genomic resource for the threatened ash species. The identification of green ash PUTs differentially expressed under stress conditions provides information for candidate gene selections that may be leveraged for future tree improvement or within genetic association or QTL studies. Our approach was to compare samples from different tissues and from different stresses to identify candidates for general stress response, being that are shared among biotic and biotic stresses. Future studies may use our publically available reference transcriptome to examine individual stresses in greater depth, and the specific molecular responses to each stress with greater statistical power. For example, we found evidence of significant transcriptional differences between EAB-susceptible and EAB–resistant green ash both prior to EAB attack and after larval feeding. This suggests that assays of additional susceptible and resistant genotypes over detailed time courses should provide important insights into mechanisms of natural resistance to EAB infestation in native ash species.

## Additional files

Additional file 1: Raw and trimmed RNASeq data. For each of the 55 green ash libraries, statistics are provided for each Illumina instrument run including: number of raw reads, number of raw bases, number of reads after trimming, number of bases after trimming, alignment rate to transcriptome. (XLSX 22 kb) Additional file 2: Incremental assemblies. For each of the nine incremental assemblies, the libraries included are listed and assembly statistics are provided for transcripts and peptides. (XLSX 39 kb) Additional file 3: Simple Sequence Repeat (SSR) information. A summary of identified SSRs is provided with additional spreadsheets listing all di-nucleotide, tri-nucelotide and tetra-nucleotide SSRs with their primer sequences. (XLSX 40 kb) Additional file 4: Differential expression results. Each differential expression experiment is included as a separate table and includes a transcript name and all output from DESeq2 including log fold change and adjusted *p*-values. Annotation information available for each transcript has been added. (XLSX 3320 kb) Additional file 5: Metastress transcript list results. A comma separated list of all transcripts belonging to stress groups. The first column contains the combination of stresses and the second column contains a tab separated list of transcripts common among that stress group. (CSV 1545 kb) Additional file 6: GO term enrichment results. Results for each GO enrichment performed are included with a corrected *p*-value for each term. (XLSX 85 kb) Additional file 7: InterProScan results. InterProScan [34] queries sequences against 16 member databases enabling protein classification by family as well as by conserved domains; the number of annotations and the percent of sequences receiving annotations varied across the different databases. (XLSX 32 kb)
